# Immunological Interactions between 2 Common Pathogens, Th1-Inducing Protozoan *Toxoplasma gondii* and the Th2-Inducing Helminth *Fasciola hepatica*

**DOI:** 10.1371/journal.pone.0005692

**Published:** 2009-05-25

**Authors:** Catherine M. D. Miller, Nicholas C. Smith, Rowan J. Ikin, Nicola R. Boulter, John P. Dalton, Sheila Donnelly

**Affiliations:** Institute for the Biotechnology of Infectious Diseases, University of Technology, Sydney, New South Wales, Australia; Federal University of São Paulo, Brazil

## Abstract

**Background:**

The nature of the immune response to infection is dependent on the type of infecting organism. Intracellular organisms such as *Toxoplasma gondii* stimulate a Th1-driven response associated with production of IL-12, IFN-γ, nitric oxide and IgG2a antibodies and classical activation of macrophages. In contrast, extracellular helminths such as *Fasciola hepatica* induce Th2 responses characterised by the production of IL-4, IL-5, IL-10 and IgG1 antibodies and alternative activation of macrophages. As co-infections with these types of parasites commonly exist in the field it is relevant to examine how the various facets of the immune responses induced by each may influence or counter-regulate that of the other.

**Principal Findings:**

Regardless, of whether *F. hepatica* infection preceded or succeeded *T. gondii* infection, there was little impact on the production of the Th1 cytokines IL-12, IFN-γ or on the development of classically-activated macrophages induced by *T. gondii*. By contrast, the production of helminth-specific Th2 cytokines, such as IL-4 and IL-5, was suppressed by infection with *T. gondii*. Additionally, the recruitment and alternative activation of macrophages by *F. hepatica* was blocked or reversed by subsequent infection with *T. gondii*. The clinical symptoms of toxoplasmosis and the survival rate of infected mice were not significantly altered by the helminth.

**Conclusions:**

Despite previous studies showing that *F. hepatica* suppressed the classical activation of macrophages and the Th1-driven responses of mice to bystander microbial infection, as well as reduced their ability to reject these, here we found that the potent immune responses to *T. gondii* were capable of suppressing the responses to helminth infection. Clearly, the outcome of particular infections in polyparasitoses depends on the means and potency by which each pathogen controls the immune response.

## Introduction

Many pathogens stimulate polarised immune responses involving the development of T helper cells with characteristic Th1 (pro-inflammatory) or Th2 (anti-inflammatory) cytokine profiles. The direction of the polarisation is often dependent on the type of infecting organism with intracellular pathogens, such as bacteria, viruses and protozoan parasites, inducing a Th1 response characterised by production of pro-inflammatory mediators such as IL-12, IFN-γ and nitric oxide and extracellular pathogens, such as helminths, inducing a Th2 response characterised by the production of IL-4, IL-5 and IL-10 [1]. A critical feature of these two types of responses is that they counter-regulate each other; thus, if one type of immune response is stimulated, the other type is suppressed [2].

A consequence of this cross regulation is that, for instance, infection with an extracellular pathogen requiring a Th2 response may inhibit a Th1 response required for control of a simultaneous intracellular pathogen. For example, infection with *F. hepatica* induces potent Th2 responses, characterised by production of IL-4, IL-5 and IL-10 [3]. At the same time, this parasite suppresses the generation of the Th1-associated cytokines, IFN-γ and IL-2 [3] and, as a result, mice co-infected with *F. hepatica* and *Bordetella pertussis* showed a significant delay in clearing the bacterial infection in the lungs [4], [5]. This modulation of the immune response was seen even though the two pathogens occupy different compartments within the body of the host, with *F. hepatica* found in the liver and *B. pertussis* confined to the lungs. The immunomodulatory effects of *F. hepatica* are systemic and begin within the first day after infection when the parasite induces the recruitment and alternative activation of macrophages; these macrophages, in turn, influence the differentiation of CD4+ T cells towards the Th2 phenotype [6], [7]. The capacity of *F. hepatica* to simultaneously down-regulate Th1 responses and up-regulate Th2 responses – and do so at the earliest time after infection - sets it apart from any other parasites studied to date [3], [6] and makes it an excellent model organism to investigate Th1/Th2 polarisation in the context of co-infection with a Th1-stimulating, intracellular infection.

Arguably, the quintessential Th1-inducing pathogen is *Toxoplasma gondii*. This protozoan parasite of humans and animals has a worldwide distribution with human infection rates of 22%–75% in different countries depending on factors such as prevalence in animals and dietary habits [8]. Acute infection is characterised by the proliferation of rapidly dividing tachyzoites that stimulate very potent Th1 cell-mediated responses stimulating the release of IL-12 from dendritic cells, neutrophils and macrophages which, in turn, activates natural killer (NK) cells to produce IFN-γ and drives proliferation of Type 1 CD4+ and CD8+ T cells, which also produce IFN-γ. IFN-γ has a major role in the development of resistance to acute infection by activating macrophages to produce nitric oxide (NO), which controls intracellular parasite growth [9], [10], [11]. This triggers stage conversion to the slow growing bradyzoite stage, contained within cysts in the skeletal muscles and central nervous system, resulting in chronic – potentially lifelong – quiescent infection. Specific immunity is required to maintain quiescence as impairment of the immune response (as seen, for example, in HIV/AIDS or organ transplants) leading to depletion of cellular immunity can result in reactivation of *T. gondii* infection and lead to the development of diseases such as toxoplasmic encephalitis [12].

The aim of this study was to investigate the developing immune responses in mice that were co-infected with a protozoan and helminth parasite; mice were infected with *T. gondii* prior and subsequent to an infection with *F. hepatica*. First, we measured isotype-specific antibody responses to the two parasites to confirm that the characteristic Th1 and Th2 responses were initiated in our co-infection model; second, the cellular compostion of peritoneal exudate cells was determined and the activation state of macrophages recovered from infected mice was determined; third, we stimulated splenocytes with *T. gondii* lysate or *F. hepatica* Excretory/Secretory (ES) antigens and measured production of the Th1 cytokine, IFN-γ, as well as the Th2-associated cytokines, IL-4 and IL-5; fourth, we compared levels of the Th1- and Th2-associated cytokines in sera and in response to non-specific stimulation of splenocytes from singly and dually-infected mice, to determine if co-infection altered production; and, fifth, we examined the effect of co-infection on the health of the host.

## Results

### Serum levels of anti-*Toxoplasma* IgG2a and anti-Fasciola IgG1 antibodies are unaffected in co-infected mice

**Figure 1:**
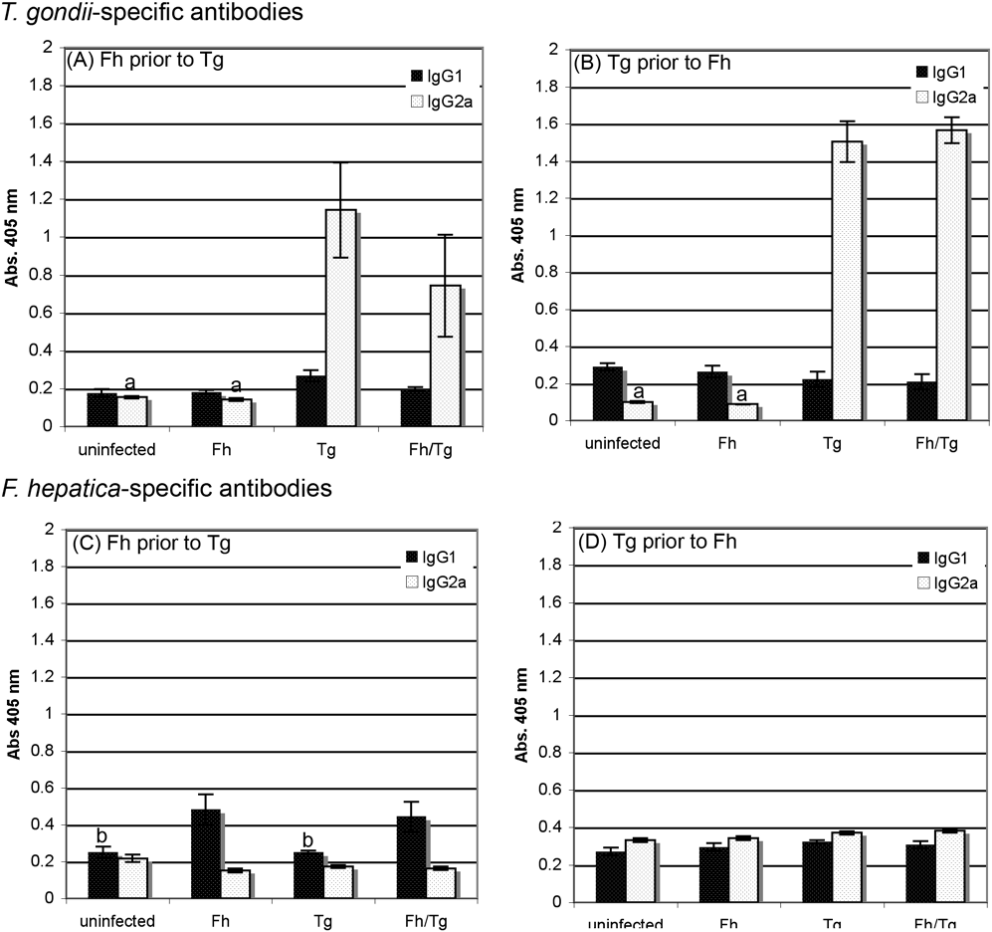
Mice infected with both *Toxoplasma gondii* and *Fasciola hepatica* produce both anti-*T. gondii* IgG2a and anti-*F. hepatica* IgG1 indicating a Th1 response to the *T. gondii* infection and a Th2 response to the *F. hepatica* infection. BALB/c mice were infected with *F. hepatica* (Fh) 5 days prior to infection with *T. gondii* (A,C) or *T. gondii* 3 days prior to infection with *F. hepatica* (B,D). Mice infected with either *F. hepatica* alone or *T. gondii* alone or uninfected mice were used as controls. Serum was collected by cardiac puncture on: (A,C) day 10 post *T. gondii* infection (day 15 post *F. hepatica* infection) or (B,D) day 14 post *T. gondii* infection (day 11 post *F. hepatica* infection). *T. gondii*-specific IgG1 and IgG2a (A,B) and *F. hepatica*-specific IgG1 and IgG2a (C,D) antibodies were measured by ELISA. The results represent the mean±SE of one experiment with five animals (A,C) or eight animals (B,D) per group. ^a^ indicates groups where the values obtained were significantly different to values obtained for the *T. gondii*-only infected mice and ^b^ represents groups where the values obtained were significantly different to those obtained for the *F. hepatica*-only infected group (*P*<0.05, Mann-Whitney non-parametric test).

Production of IgG1 has been associated with a developing Th2 response whereas IgG2a has been associated with a Th1 response, so production of both isotypes was examined in the co-infected mice to establish what type of response was initiated. Infection with *T. gondii* induced high levels of specific IgG2a antibody, with little or no IgG1; the absorbance values for IgG2a were not significantly different between mice infected with *T. gondii* alone or with this parasite and *F. hepatica*. Similar results were seen regardless of the order of infection (Fig. 1A and B). In contrast, no antigen-specific IgG2a was detected in any of the groups infected with this *F. hepatica* regardless of order of infection (Fig. 1C and D); however, when *F. hepatica* was the first infecting parasite it induced potent specific IgG1 responses that were not ameliorated by co-infection with *T. gondii* (Fig. 1C), showing that a Th2 response had been induced, at least initially, in co-infected mice. No *F. hepatica*-specific IgG1 antibody was detected in any of the groups when mice were infected with *T. gondii* before *F. hepatica* (Fig. 1D).

### *Toxoplasma gondii* suppresses the recruitment and alternative activation of macrophages normally associated with helminth infection

**Figure 2:**
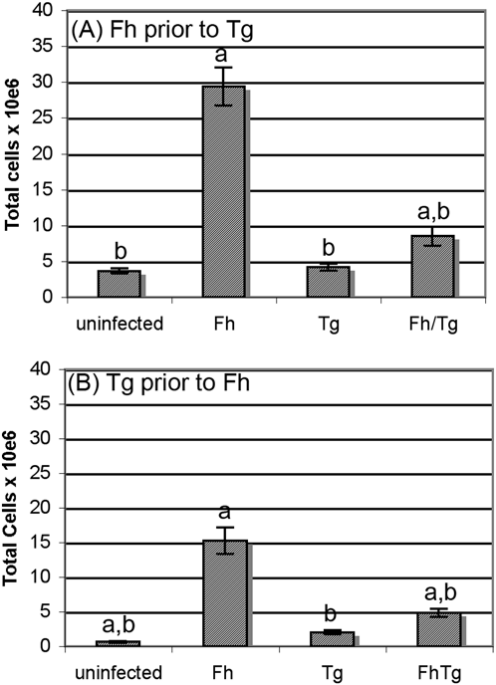
*Toxoplasma gondii* infection suppresses the recruitment of cells to the site of infection normally associated with *F. hepatica* infection. (A) BALB/c mice were infected with *F. hepatica* (Fh) then 5 days later were infected with *T. gondii* (Tg) or (B) mice were infected with *T. gondii* 3 days prior to infection with *F. hepatica*. Mice infected with either *F. hepatica* alone or *T. gondii* alone or uninfected mice were used as controls. On days 10 (A) and 14 (B) post infection *T. gondii* (days 15 and 11 post *F. hepatica* infection respectively) peritoneal exudate cells were recovered from the peritoneal cavity by flushing with PBS. Total cell numbers were determined by counting using a Neubauer slide. Microscopic analysis showed macrophages to be the dominant cell type in all the mice making up 89–91% of cells; other cells present included lymphocytes (4–5%) and neutrophils (5–6%). Results are presented as mean±SE for five animals per group (A) or eight animals per group (B). ^a^ represents groups where the values obtained were significantly different to values obtained for the *T. gondii*-only infected mice and ^b^ represents groups where the values obtained were significantly different to those obtained for the *F. hepatica*-only infected group (*P*<0.05, Mann-Whitney non-parametric test).

A feature of infections by helminths such as *F. hepatica* is the recruitment of large numbers of cells to the site of infection [2]. The numbers of peritoneal exudate cells (PEC) in mice infected with both *F. hepatica* and *T. gondii* were significantly lower than the numbers found in *F. hepatica*-only infected mice on day 15 of *F. hepatica* infection (day 10 of *T. gondii* infection; *P*<0.05; Fig. 2A). The numbers of PEC in these co-infected mice were slightly, but significantly, higher than the numbers of PECs found in *T. gondii*-infected and uninfected mice (*P*<0.05; Fig. 2A). Similar results were seen when *T. gondii* infection preceded *F. hepatica* infection. The recruitment of cells is still obvious despite the earlier time point (day 11 post *F. hepatica* infection) but this has been significantly reduced in co-infected mice (*P*<0.05; Fig. 2B). Again, the numbers of PEC in the co-infected mice were slightly, but significantly, higher than the numbers of PECs found in *T. gondii*-infected and uninfected mice (*P*<0.05; Fig. 2B). Microscopic analysis showed macrophages to be the dominant cell type in all the mice making up 89–91% of cells; other cells present included lymphocytes (4–5%) and neutrophils (5–6%). As is typical for mice infected with *F. hepatica* (in contrast to other animal models of *F. hepatica* infection), no eosinophilia was observed [5].

Macrophage activation status was assessed to determine whether they were classically or alternatively activated. Classically activated macrophages express iNOS, which catalyses the metabolism of arginine into nitric oxide, and produce the proinflammatory cytokine, IL-12. Conversely, alternatively activated macrophages express Arginase 1 that catabolises the conversion of arginine into creatine phosphate [6], [13].

**Figure 3:**
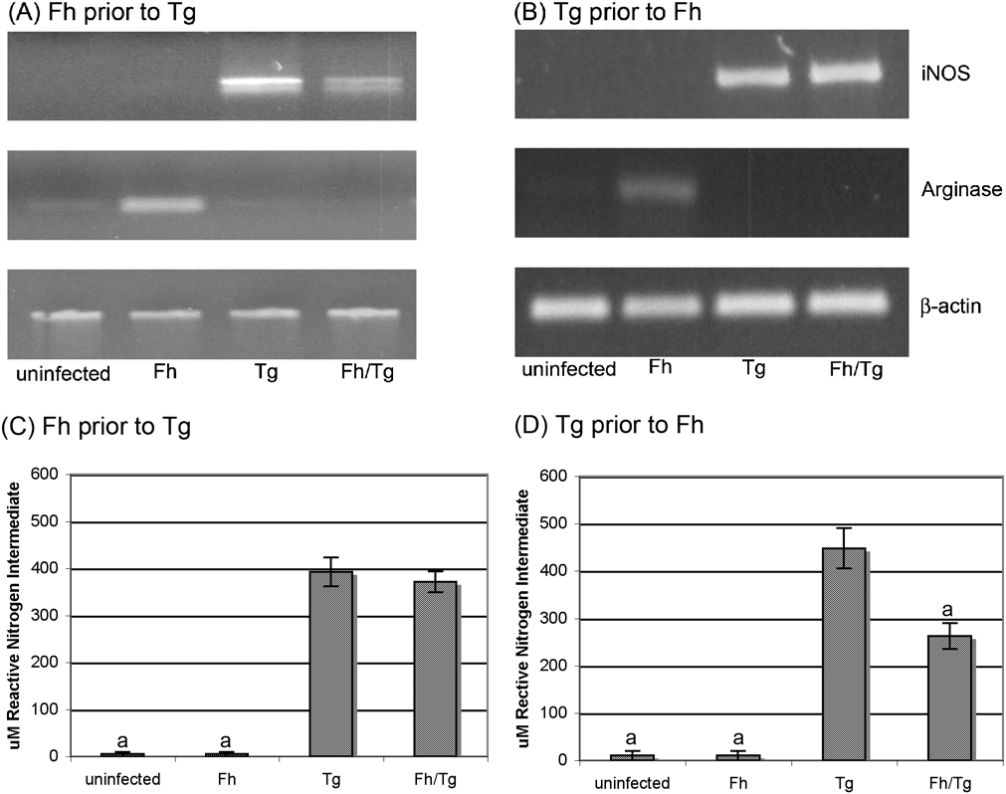
Macrophages recovered from the peritoneal cavity of mice co-infected with *T. gondii* and *F. hepatica* are classically activated. Mice were infected with *F. hepatica* 5 days prior (A) or 3 days subsequent (B) to infection with *T. gondii*. Mice infected with either *F. hepatica* alone or *T. gondii* alone were used as controls. Fifteen (A) or eleven (B) days post *F. hepatica* infection (day 10 (A) or 14 (B) post *T. gondii* infection respectively) peritoneal exudate cells were recovered from the peritoneal cavity by flushing with PBS. Expression levels of inducible nitric oxide synthase (iNOS), Arginase 1 (Arg 1) and β-actin in adhered peritoneal exudate cells were analysed by RT-PCR. The data shown are from single mice and are representative of results obtained from all mice in each group. Serum levels of NO were determined in the same mice. Serum was collected by cardiac puncture on (A) day 10 post *T. gondii* infection (day 15 post *F. hepatica* infection) or (B) day 14 post *T. gondii* infection (day 11 post *F. hepatica*). A modified Griess reaction was used to measure NO (see Materials and Methods). The results represent the mean±SE of one experiment with five animals per group (A) or eight animals per group (B). ). ^a^ represents groups where the values obtained were significantly different to values obtained for the *T. gondii*-only infected mice and ^b^ represents groups where the values obtained were significantly different to those obtained for the *F. hepatica*-only infected group (*P*<0.05, Mann-Whitney non-parametric test).

Macrophage activation was first evaluated by monitoring expression of genes coding for iNOS and Arginase I using RT-PCR. Macrophages from mice infected with *F. hepatica* alone exhibited elevated expression of Arginase 1 but not iNOS, indicating their alternative activation. In contrast, macrophages from co-infected and from those infected with *T. gondii* alone expressed iNOS but not Arginase 1, indicating that the cells were classically activated. This pattern was observed whether *F. hepatica* was the first infecting organism (Fig. 3A) or the second (Fig. 3B). Neither iNOS nor Arginase I was expressed in macrophages from uninfected mice (Fig. 3A, B).

Consistent with the RT-PCR results, adherent peritoneal macrophages from *T. gondii*-only and co-infected mice, left in culture for 48 hours without any antigenic stimulus, spontaneously produced reactive nitrogen intermediates (469.1±65.5 µM and 498.3±81.7 µM nitrate, respectively, mean±S.E., n = 5 mice) whereas cells from uninfected mice or mice infected only with *F. hepatica* produced negligible levels of NO. Serum NO levels (at 10 or 14 days post *T. gondii* infection) confirmed these results and, thus, were elevated in mice acutely infected with *T. gondii* but remained at background levels in mice infected with *F. hepatica* only. Hence, in mice infected with *F. hepatica* prior to infection with *T. gondii*, NO levels on day 10 post *T. gondii* infection were not significantly different from those seen in mice infected with *T. gondii* alone (Fig. 3C). In mice infected with *F. hepatica* subsequent to infection with *T. gondii*, NO levels on day 14 post *T. gondii* infection were significantly reduced compared with levels in mice infected with *T. gondii* alone but were still significantly higher than the levels seen in mice infected with *F. hepatica* alone or naïve mice (Fig. 3D).

**Figure 4:**
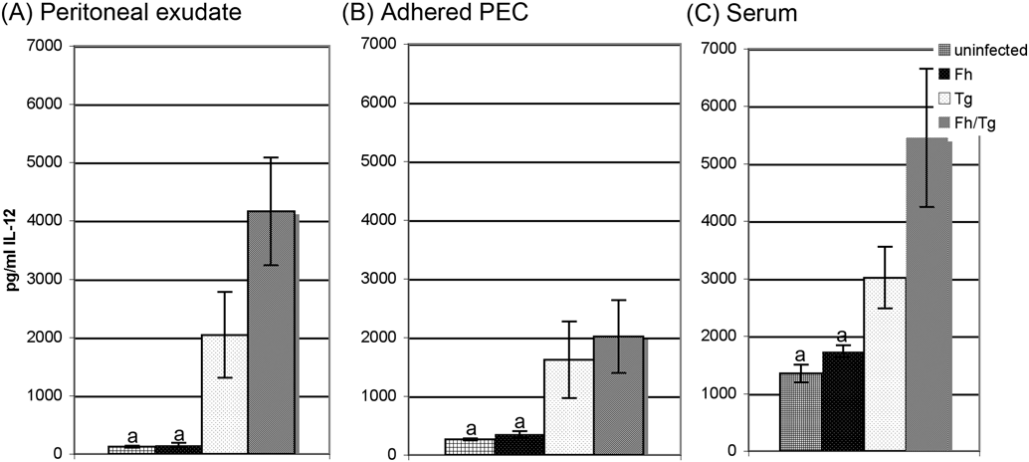
Infection with *Fasciola hepatica* does not inhibit IL-12 production early in infection with *Toxoplasma gondii.* BALB/c mice were infected with *F. hepatica* 5 days prior to infection with *T. gondii*. Mice infected with either *F. hepatica* alone or *T. gondii* alone were used as controls. On day 4 post *T. gondii* infection (day 9 post *F. hepatica* infection) the peritoneal cavity was rinsed with PBS and the resulting lavage was spun at 500 *g* to pellet the exudate cells. The supernatant was retained to measure the level of IL-12 being secreted into the peritoneal cavity by cytokine ELISA (B). Adherent peritoneal exudate cells (PEC) were cultured for 48 h and supernatants assayed for secretion of IL-12 by cytokine ELISA (A). Serum was collected by cardiac puncture and levels of IL-12 determined by cytokine ELISA (C). The results represent the mean±SE of one experiment with five animals per group. ^a^ represents groups where the values obtained were significantly different to values obtained for the *T. gondii*-only infected mice and ^b^ represents groups where the values obtained were significantly different to those obtained for the *F. hepatica*-only infected group (*P*<0.05, Mann-Whitney non-parametric test).

Confirmation of the classical activation status of the macrophages from co-infected mice was also sought by analysing IL-12 production by peritoneal macrophages early after infection with *T. gondii*. Thus, 4 days after infection with *T. gondii*, levels of IL-12 in peritoneal exudate and spontaneous secretions from adhered peritoneal exudate cells were significantly elevated in co-infected mice compared with levels detected in *F. hepatica*-infected and uninfected mice, but were not significantly different from the levels seen in *T. gondii*-infected mice (Fig. 4A, B). This pattern was reaffirmed in sera, where IL-12 levels were elevated significantly above background levels in mice infected only with *T. gondii* and increased further in mice infected with both *T. gondii* and *F. hepatica* (Fig. 4C).

### Production of *T. gondii*-specific Th1 cytokines is not suppressed by *F. hepatica* infection but *T. gondii* infection inhibits the ability of splenocytes from Fasciola-infected mice to produce Th2 cytokines

**Figure 5:**
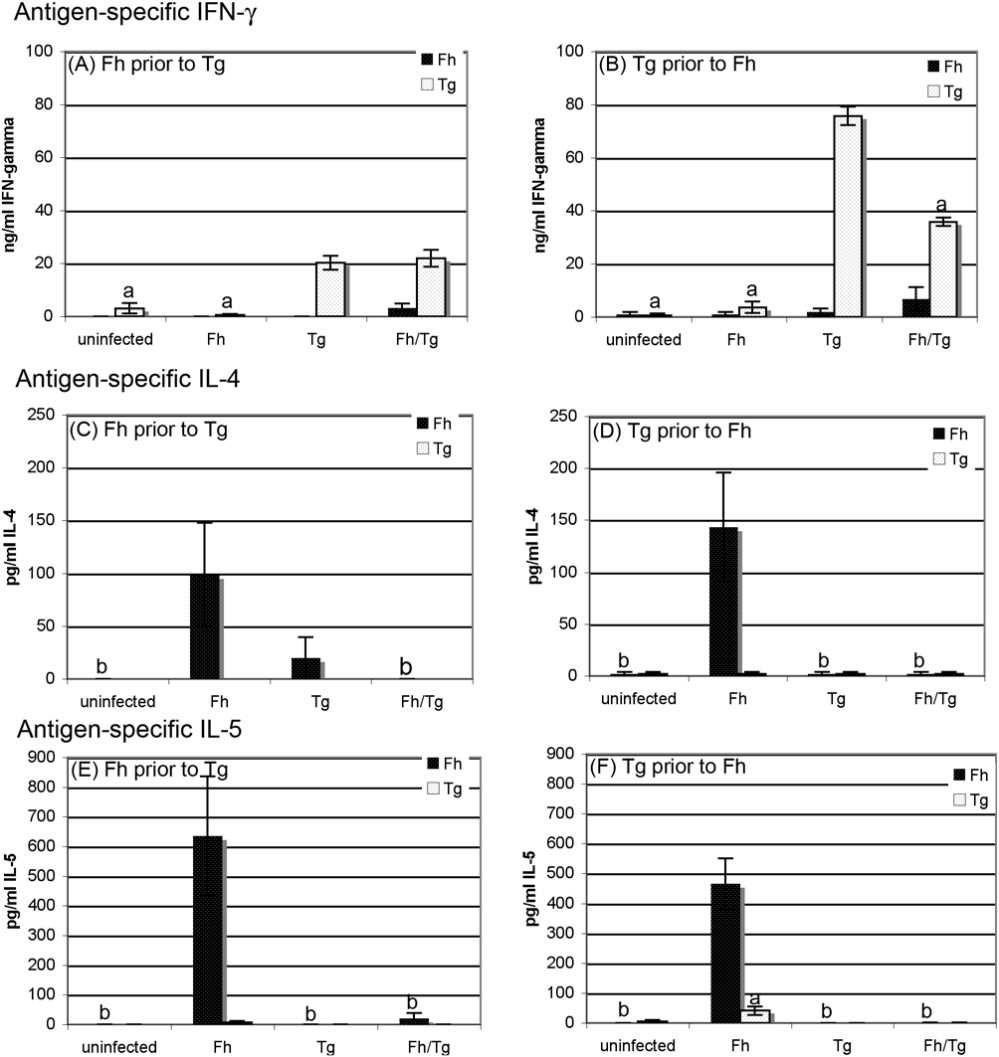
Production of *T. gondii*-specific Th1 cytokines is not suppressed by *F. hepatica* infection but *T. gondii* infection inhibits the ability of splenocytes from *F. hepatica*-infected mice to produce Th2 cytokines. Mice were infected with *F. hepatica* 5 days prior or 3 days subsequent to infection with *T. gondii*. Mice infected with either *F. hepatica* alone or *T. gondii* alone were used as controls. Cytokine production of the Th1-associated cytokine IFN-γ (A, B) and the Th2-associated cytokines, IL-4 (C, D) and IL-5 (E, F) by spleen cells was assessed on days 10 and 14 post *T. gondii* infection (day 15 or 11 post *F. hepatica* infection respectively) by stimulation *in vitro* with ES antigen or *T. gondii* lysate. Cytokine concentrations represent mean±SE after subtraction of background control values with medium only for five mice (A, C, E) or eight mice (B, D, F) per group. ^a^ indicates groups where the values obtained were significantly different to values obtained for the *T. gondii*-only infected mice and ^b^ represents groups where the values obtained were significantly different to those obtained for the *F. hepatica*-only infected group (*P*<0.05, Mann-Whitney non-parametric test).

Stimulation of splenocytes with *T. gondii* antigen resulted in the production of significant amounts of *T. gondii*-specific IFN-γ in mice infected with *T. gondii* only and in co-infected mice compared with uninfected mice or mice infected with *F. hepatica* only, regardless of order of infection (*P*<0.05; Fig. 5A, B). When mice were infected with *F. hepatica* prior to infection with *T. gondii*, there was no significant difference in levels of *T. gondii*-specific IFN-γ between co-infected mice and mice infected with *T. gondii* only (*P*>0.05; Fig. 5A). When mice were infected with *F. hepatica* subsequent to infection with *T. gondii*, there was a slight, but statistically significant, reduction in the levels of *T. gondii*-specific IFNγ in co-infected mice compared with mice infected with *T. gondii* only (*P*<0.05; Fig. 5B) but the level of IFN-γ produced was still relatively high (at a mean of >30 ng/ml, it was actually higher than the values seen in samples taken from mice infected with *F. hepatica* prior to *T. gondii*, where the mean was just over 20 ng/ml).

*T. gondii* antigen did not stimulate production of *T. gondii*-specific IL-4 or IL-5 in splenocytes from any group of mice but stimulation with *F. hepatica* ES products resulted in the production of high levels of these cytokines in mice infected with *F. hepatica* alone. Most strikingly, production of *F. hepatica*-specific IL-4 and IL-5 was absent in mice concurrently infected with *T. gondii* and *F. hepatica* regardless of whether *T. gondii* was the first infecting organism (Fig. 5C, E) or the second (Fig. 5D, F).

**Figure 6:**
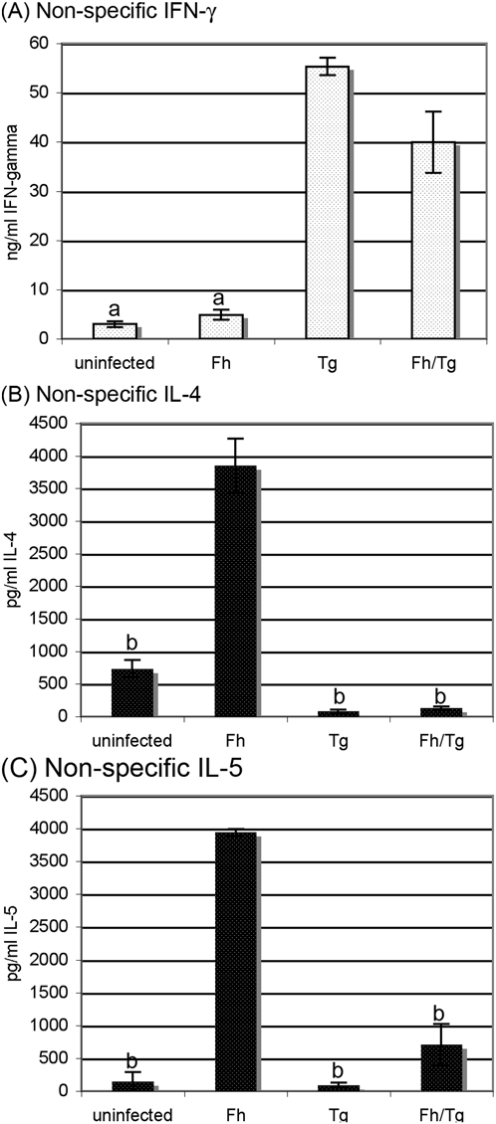
*Toxoplasma gondii* suppresses *Fasciola hepatica*-driven non-specific Th2 responses in co-infected mice. Mice were infected with *F. hepatica* 5 days prior to infection with *T. gondii* to allow time for a Th2 response to be induced. Mice infected with either *F. hepatica* alone or *T. gondii* alone were used as controls. Fifteen days post *F. hepatica* infection (day 10 post *T. gondii* infection) spleen cells were stimulated *in vitro* with PMA plus anti-CD3 and medium alone was included as a negative control. Levels of the Th1-associated cytokine IFN-γ (A) and the Th2-associated cytokines, IL-4 and IL-5 (B, C) were assessed in supernatants 3 days later. Cytokine concentrations represent mean±SE after subtraction of background control values with medium only for five mice per group. ^a^ represents groups where the values obtained were significantly different to values obtained for the *T. gondii*-only infected mice and ^b^ represents groups where the values obtained were significantly different to those obtained for the *F. hepatica*-only infected group (*P*<0.05, Mann-Whitney non-parametric test).

The effects of *T. gondii* were also apparent when splenocytes were non-specifically stimulated. Thus, cytokine production in response to stimulation with PMA and anti-CD3ε showed a clear polarisation towards a Th1 response in co-infected mice, with a suppression of *F. hepatica*-driven Th2 responses in co-infected mice. Hence, there was no significant difference in the levels of IFN-γ produced by splenocytes from co-infected mice compared with *T. gondii*-infected mice (*P*>0.05; Fig. 6A) but levels of IL-4 and IL-5 secretion by splenocytes from co-infected mice were significantly lower than the levels from *F. hepatica*-infected mice (*P*<0.05; Fig. 6B,C). These results were reflected in sera of infected mice. Whilst not detectable in sera from uninfected or *F. hepatica*-only infected mice, IFN-γ levels in sera from co-infected mice (19.4+1.6 ng/ml, mean±S.E., n = 8 mice) were not different from *T. gondii*-only infected mice (20.6±3.7 ng/ml, n = 8). At the same time, IL-4 was not detectable in sera from uninfected mice, *T. gondii*-only or co-infected mice, but was readily detected in the sera of *F. hepatica*-only infected mice (31±10 pg/ml, n = 8). Likewise, IL-5 was not detected in sera from uninfected or *T. gondii*-only infected mice but was elevated in sera from *F. hepatica*-only infected mice (159.4±26.9 pg/ml, n = 8) and significantly lower (*P*<0.05) in co-infected mice (65.4±45.2 pg/ml, n = 8).

### Co-infection with *Fasciola hepatica* has little effect on the course of acute *T. gondii* infection

We observed no difference in severity of clinical signs such as ruffled fur and hunching in co-infected mice compared with *T. gondii*-infected mice. There was no obvious increase in liver pathology in co-infected mice compared with *F. hepatica*-infected mice based on the visual assessment of the whole liver and examination of haematoxylin and eosin stained sections. Livers from both groups of animals showed tracks of necrotic areas caused by the migration of the juvenile flukes. Between 3–5 flukes were seen in both single-infected and dual-infected livers. No large areas of necrosis were seen in livers from *T. gondii*-infected or uninfected mice. When *F. hepatica* infection preceded *T. gondii* infection, the overall survival rate of co-infected mice was affected slightly −88% of mice (15/17) used over the course of three experiments survived co-infection compared with 100% survival in mice infected with *T. gondii* (n = 17) or *F. hepatica* only (n = 15). However, when *T. gondii* infection preceded *F. hepatica* infection, there was no effect on survival rate with all co-infected mice (n = 14) surviving until the end of the experiment.

## Discussion

*Fasciola hepatica* and *Toxoplasma gondii* are prime examples of extracellular helminth and intracellular protozoan parasites that induce Th2- and Th1-biased immune responses, respectively. Our detection of IgG1-specific antibodies to *F. hepatica* and IgG2a-specific antibodies to *T. gondii* confirms this ability. Moreover, the fact that levels of IgG1 induced by *F. hepatica* and levels of IgG2a induced by *T. gondii* were similar in singly infected and co-infected mice, at least when *F. hepatica* infection preceded *T. gondii* infection (Fig. 1A), is evidence that both parasites stimulated their own distinctive Th-cell subset, even in co-infections. Most importantly, these antibody results indicate that *F. hepatica* initially stimulated a Th2-biased response in our co-infection experiments and, thus, the classical activation state of macrophages and Th1-biased cytokine profiles we observed in co-infected mice reflect an alteration of the established *F. hepatica*-induced Th2-biased response by *T. gondii*. The absence of *F. hepatica*-specific IgG1 antibodies in co-infected mice where *T. gondii* infection preceded *F. hepatica* infection (Fig. 1B) possibly confirms this notion but some qualification of this statement is required because, in this experiment, sera were collected on day 11 post *F. hepatica* infection, which may be too soon to detect *F. hepatica*-specific IgG1.

A large cellular infiltrate, predominantly macrophages, to the site of infection is a central manifestation of helminth infections such as, for example, *F. hepatica* [2] and *Brugia malayi* [14]. Consistent with previous studies [6] we observed that *F. hepatica* stimulates the recruitment of macrophages into the peritoneal cavity within the first few days of infection. However, we found that *T. gondii* significantly reduced the recruitment of macrophages to the peritoneum in mice previously infected with *F. hepatica*. A similar result was seen in the experiment whereby mice were infected with *T. gondii* prior to infection with *F. hepatica* suggesting that *T. gondii* affects the innate chemotactic responses that result in the cell infiltration. While T-cell derived chemokines promote this cellular recruitment, it is believed that innate immune signals may initiate cell mobilization that is then maintained by established adaptive immune responses [15]. Indeed, Th2-associated alternatively activated macrophages express specific chemokines [16] that preferentially attract Th2 cells [17]. Given the Th1 bias of *T. gondii* infection it is of interest to note that expression of these Th2 related chemokines is down-regulated by Th1 associated cytokines such as IFNγ and TNFα [17], [18] and is not detected in Th1 associated infections such as *Staphylococcus aureus*, *Candida albicans*, or influenza virus [18].

Macrophages recruited to the peritoneum by *F. hepatica* show all the hallmarks of alternative activation; by 5 days after infection, the expression of markers of alternative activation, Fizz1, Arginase 1 and Ym1 are up-regulated and remain so for the subsequent 3 weeks [6]. In this study, we have shown that macrophages recovered from *F. hepatica*-infected mice are alternatively activated, however, macrophages from mice co-infected with *T. gondii* were shown to be classically activated, similar to those found in mice infected with *T. gondii* alone. This was reflected in the secretion of nitric oxide and pro-inflammatory cytokines including IL-12 by adherent peritoneal macrophages, in peritoneal exudate and in elevated levels in sera. Therefore, the idea that alternatively activated macrophages play a critical role in the development of Th2 responses by helminth parasites [6], [7], [14], [19] is supported by our data showing that *T. gondii* also downplayed the development of antigen-specific Th2 responses to *F. hepatica*. While the appropriate Th1 or Th2 response was observed in splenocytes taken from the singly-infected animals, the *F. hepatica*-specific Th2 response in co-infected animals was overwhelmed by the *T. gondii*-specific Th1 response (Fig. 5).

The dominant Th1-biased response induced by *T. gondii* was also seen at a non-specific level. Thus, non-specific T-cell responses (to PMA and anti-CD3ε) induced by *F. hepatica* were switched from a Th2 bias to a Th1-predominant response by *T. gondii* although the effect was more obvious where *F. hepatica* infection preceded *T. gondii* infection than the other way around (Fig. 6). Serum levels of typical Th1 and Th2 cytokines and immune effectors were consistent with this modulation of non-specific cytokine responses. Thus, the levels of the inflammatory mediators associated with a Th1 response dominated in co-infected mice and were similar in the sera of mice infected with *F. hepatica* prior to infection with *T. gondii* and mice infected with *T. gondii* only.

Previous studies from our laboratory showed that infection with *F. hepatica* suppressed the production of Th1 cytokines to a bystander infection with the pulmonary microbe *B. pertussis*, and was sufficiently strong to reduce the ability of the mice to clear the bacterium [4], [5]. The parasite was also capable of suppressing the Th1 response to a *B. pertussis* whole-cell vaccine [4]. It is interesting, therefore, that in this study this helminth did not exhibit any suppression of responses to *T. gondii*. However, these results are in keeping with those of others that demonstrated the ability of *T. gondii* to potently regulate the host's immune response. For example, *T. gondii* reduces the Th2 responses induced by helminth infections such as *Nippostronglylus brasiliensis* [20] and *Schistosoma mansoni* [21], enhances Th1 responses to *Leishmania major* infection [22], shifts Th2 to Th1 immunity during chronic *H. felis* [23] infection and elevates Th1 responses to non-related antigens [24]. An important difference in our model is that *F. hepatica* is, to date, the only infectious agent that simultaneously promotes Th2 responses and inhibits Th1 responses at the earliest time after infection, and the Th1-biased response to *T. gondii* still ensued.

In keeping with the lack of effect of *F. hepatica* on the Th1 response to *T. gondii*, there was no significant effect on clinical symptoms of toxoplasmosis in co-infected mice. Co-infection of mice with *F. hepatica* and *T. gondii* did, however, appear to adversely affect their survival, albeit slightly; 88% of the co-infected mice in our study survived infection for at least the 15 days of the experiments, however, all mice infected with *F. hepatica* or *T. gondii* alone survived. This represents only a couple of infected mice and, whether it is a biologically significant result or not would require experimentation on a much larger number of animals. Thus, whether this loss of life is due to an enhanced susceptibility to *T. gondii* as a result of the pre-existing *F. hepatica* infection is impossible to say but appears unlikely since mice did not exhibit any of the classical outward symptoms associated with fatal acute infection with *T. gondii*. Likewise, enhanced susceptibility to *F. hepatica* as a result of infection with *T. gondii* is difficult to ascribe; there was no enhancement of gross liver pathology in the co-infected mice and no difference in the number of parasites harboured by the co-infected and singly-infected groups. Nevertheless, the possibility that liver function was disrupted in the co-infected mice cannot be dismissed, particularly in light of a previous study on co-infection of mice with *T. gondii* and another fluke, *S. mansoni*, where death of the hosts was convincingly ascribed to liver dysfunction associated with production of TNF and IL-12 [21], [25]. Furthermore, the presence of macrophages secreting reactive nitrite intermediates such as nitric oxide can be readily imagined to contribute to the slight increase in susceptibility to infection; nitric oxide is known to contribute to the pathology seen in infection through its damaging action on cell membranes [26]. This would perhaps have little effect on juvenile flukes since they are resistant to the actions of free radicals such as nitric oxide [27] but could have had damaging effects on the organs in the peritoneal cavity. Thus, (grossly inapparent) effects of nitric oxide-mediated damage combined with the pathology caused by the migration of *F. hepatica* through the liver may have contributed to the increased susceptibility seen in the co-infected mice.

In summary, our results confirm that the intracellular pathogen *T. gondii* stimulates a strong Th1 response characterised by the early classical activation of macrophages and the production of the inflammatory mediators IL-12, IFN-γ and NO. These responses are sufficiently potent as to suppress the development of alternative activation of macrophages and, thus, the Th2 responses associated with a pre-established infection with the helminth *F. hepatica*. Given these results and the ubiquity of *T. gondii* infection, it seems to us that the influence and importance of this parasite on animal and human health may be severely under-appreciated, particularly as a modulator of immune responsiveness in co-infections.

## Materials and Methods

### Parasites and infections

All animal research was performed with the approval of the UTS/Royal North Shore Hospital Animal Care & Ethics Committee. Tachyzoites of the *T. gondii* Me49 strain were obtained from the ATCC and subsequently maintained by continuous passage in Vero cells (using RPMI [Gibco] and 2% heat inactivated newborn calf serum [NBS]) at 37°C in a 5% CO_2_ incubator. Parasites were harvested from freshly lysed cultures, passed through a 26G needle and concentrated by spinning at 500 *g*. The pellet of tachyzoites was resuspended in sterile 0.9% saline. Parasites were counted using a Neubauer haemocytometer and diluted in sterile 0.9% saline to the required dose for injection. *T. gondii* lysate was prepared by resuspending tachyzoites in PBS, sonicating for 3×10 sec at 50 W/20 kHz and centrifuging at 13,000 *g* to remove insoluble debris. Metacercariae and adult worms of *F. hepatica* were obtained from the Elizabeth Macarthur Agricultural Institute, N.S.W. Department of Agriculture (Camden, Australia). Excretory-secretory (ES) products were prepared by incubating adult worms in culture medium for 24 h as previously described [6]. Protein concentrations were determined using the Lowry protein assay (Biorad dye reagent).

A pilot experiment was conducted where 6- to 8-weeks old female BALB/c mice were infected orally with approximately 20 metacercariae of *F. hepatica* 2 days prior to, immediately after and 2 days subsequent to *T. gondii* infection (achieved by i.p. injection of 250 tachyzoites, after previous dose response experiments demonstrated that, in our hands, this number of tachyzoites was optimal for inducing Th1 responses in mice). Mice were euthanased on day 10 post *T. gondii* infection and a limited number of immunological parameters assessed. Data from this experiment and from previous experiments that were not part of this study were used to set time points for larger, more in depth experiments.

We chose the i.p. injection of tachyzoites for our model for two main reasons. First, estimation of the infective dose of tachyzoites is far more reliable that that of bradyzoites since each tissue cyst contains different numbers of tachyzoites and, furthermore, the dissemination patterns following oral infection of mice with tissue cysts is inconsistent [28]. Second, we wanted to establish a rapid, predictable and reproducible response in the peritoneal cavity, which is the early site for infection with juvenile fluke, and this is best achieved using i.p. injection of tachyzoites though, ultimately, both infection routes result in similar responses [29]; this was particularly important in relation to our observations on recruitment and activation of peritoneal macrophages in response to *F. hepatica* and in co-infected mice.

To examine the effect of *T. gondii* on *F. hepatica*-induced Th2 responses fifteen 6- to 8-weeks old female BALB/c mice (Gore Hill Research Laboratories, Sydney, Australia) were infected orally with *F. hepatica* 5 days prior to infection with *T. gondii*. Five mice per day were euthanased on days 4, 7 or 10 post *T. gondii* infection (i.e. 9, 12 and 15 days after *F. hepatica* infection). In a reverse experiment, sixteen 6- to 8-week old mice were infected with *T. gondii* 3 days prior to infection with *F. hepatica*. Eight mice per day were euthanased on day 10 and 14 post *T. gondii* infection (i.e. 7 and 11 days after *F. hepatica* infection). Controls included mice infected with *T. gondii* and *F. hepatica* only and naive mice. Similar results were achieved between the pilot study and the larger studies.

Mice were monitored daily for clinical signs of acute *T. gondii* infection - ruffled fur, hunched posture, lethargy and morbidity - before being euthanased by terminal anaesthesia. The livers of all animals were removed and assessed visually for signs of necrosis caused by migrating *F. hepatica*. Damage to livers was assessed on a scale of 1 to 5 with 1 meaning no visible damage and 5 meaning heavy damage. Sections of formalin-fixed and wax embedded liver were examined for areas of necrosis following staining with haematoxylin and eosin (H&E).

### Isolation and characterisation of peritoneal exudate cells (PEC)

PECs were recovered by washing the peritoneal cavity with 6 ml of sterile phosphate-buffered saline (PBS). Total cell numbers of PECs were estimated using a Neubauer hemocytometer and cell types were analysed after staining with May-Grünwald's solution and Giemsa's solution (Merck). The remaining cells were centrifuged at 500 *g*, the supernatant retained for cytokine analysis, while the pelleted cells were resuspended in RPMI 1640 containing 10% NBS and cultured in 6 well plates at 37°C/5%CO_2_. After 2–3 h incubation, non-adhered cells were removed by washing with RPMI and adhered cells (mainly macrophages) were removed by scraping. Cells were counted, adjusted to a concentration of 1×10^6^/ml and replated into 48 well plates. Cells were incubated at 37°C/5%CO_2_ for 48 h before supernatants were removed and stored at −20°C until analysis. Supernatants were assayed for cytokines and NO as described below. Cells were recovered for RNA extraction and RT-PCR by resuspending in Tri-reagent (Sigma) added directly to the well. Samples were stored at −70°C until analysis.

For RT-PCR, first strand cDNA was produced with oligo dT primers from total RNA using AMV reverse transcriptase (Promega) at 42°C for 60 min. Aliquots of the resulting cDNA were amplified using primers specific for β-actin and Arginase 1 [30] and inducible nitric oxide synthase [iNOS; 31] under the following conditions: 30 s denaturation at 95°C, annealing of primers at 56°C for 5 sec, and 12 sec elongation at 72°C for 40 cycles. All PCR products were electrophoresed on 1% agarose gels and visualised by ethidium bromide staining.

### Immunological assays

*T. gondii-* and *F. hepatica*-specific IgG1 and IgG2 in serum was measured by ELISA [32]. Briefly, 96 well microtiter plates (Nunc) coated with 1 µg/well of *F. hepatica* ES products or *T.gondii* Me49 lysate diluted in 0.1 M carbonate buffer were incubated with serum diluted 1∶100 in 0.05% bovine haemoglobin in 0.3% Tween in PBS. Single dilution assays were carried out where reference samples were used to take into account plate-to-plate and day-to-day variation as recommended in a detailed analysis of the topic by Venkatesan and Wakelin [33]. Bound antibody was detected using biotinylated anti-mouse IgG1 and IgG2a antibodies (BD Pharmingen, San Diego, CA), ExtrAvidin-alkaline phosphatase conjugate and ρ-nitrophenyl phosphate as substrate.

Splenocytes were cultured at 5×10^6^ cells/ml with 50 µg/ml of *T. gondii* soluble extract or *F. hepatica* ES products. Control stimuli were medium alone or anti-CD3ε (2 µg/ml) plus phorbol myristate acetate (PMA; 25 ng/ml). Concentrations of IL-12, IFN-γ, IL-4 and IL-5 were measured by immunoassay using matched pairs of anti-cytokine antibodies purchased from BD Pharmingen (San Diego, CA) according to the manufacturer's instructions. Recombinant cytokines of known concentration were used to generate standard curves.

NO produced by the adhered PEC was measured by mixing equal amounts of supernatant with Griess reagent (1% sulfanilimide in 2.5% H_3_PO_4_ and 0.1% napthylethylenediamine dihydrochloride in 2.5% H_3_PO_4_ mixed in a 1∶1 ratio just prior to assay). Absorbance was measured at 540 nm and the amount of nitrite was determined by comparing against a standard curve. Serum NO concentrations were determined as described previously [34]. Briefly, serum nitrate was converted to nitrite using 5 U/ml nitrate reductase (Sigma) and 1.25 mg/ml nicotinamide adenine dinucleotide phosphate (NADPH; Sigma) and the amount of nitrite determined using Griess reagent as described above. Serum nitrite was also determined by mixing serum directly with Griess reagent. Results were expressed as micromolar concentrations of reactive nitrogen intermediates (RNI).

### Statistical analyses

The statistical significance of differences between groups was determined using a Kruskal-Wallis non-parametric test. Pairs of groups were also compared using a Mann-Whitney non-parametric test. A *P* value of <0.05 was considered significant for both tests.
